# The characterization and expression analysis under stress conditions of *PCST1* in *Arabidopsis*

**DOI:** 10.1080/15592324.2022.2134675

**Published:** 2022-10-25

**Authors:** Hao Zhang, Ke Zhang, Tongtong Liu, Ying Zhang, Ziyan Tang, Jingao Dong, Fengru Wang

**Affiliations:** a State Key Laboratory of North China Crop Improvement and Regulation, Hebei Key Laboratory of Plant Physiology and Molecular Pathology, College of Life Sciences, Hebei Agricultural University, Baoding, China; b State Key Laboratory of North China Crop Improvement and Regulation, College of Agronomy, Hebei Agricultural University; Key Laboratory of Crop Growth Regulation of Hebei Province, Baoding, China; c Pear Engineering and Technology Research Center of Hebei, College of Horticulture, Hebei Agricultural University, Baoding, China; d College of Plant Protection, Hebei Agricultural University, Baoding, China

**Keywords:** PCST1, START domain, gene function, *arabidopsis*

## Abstract

Analysis of *PCST1* expression characteristics and the role of *PCST1* in response to osmotic stress in *Arabidopsis thaliana*. The structure of *PCST1* was analyzed using Bioinformatics method. Real-time PCR, GUS tissue localization and subcellular localization were adopted to analyze the expression pattern of *PCST1* in Arabidopsis. To validate the transgenic positive strain of *PCST1* using Real-time PCR, overexpression experiments were performed in wild type. Full-length cDNA was cloned and connected into a binary vector with 35S promoter, and the construction was transformed into wild type. With NaCl and mannitol treatments, the germination rate, green leaves rate, physiological indexes were carried out and counted in Arabidopsis with overexpression of *PCST1* and T-DNA insertion mutants. The molecular mechanism of *PCST1* in response to osmotic stress in Arabidopsis was analyzed. Based on the bioinformatic analysis, PCST1 is a hydrophobin with 403 amino acids, and the molecular weight is 45.3236 KDa. It contains only the START (the lipid/sterol – binding StAR – related lipid transfer protein domains) conservative domain. PCST1 possesses phosphatidylcholine binding sites and transmembrane region. Expression pattern analysis showed that expression of *PCST1* increased with time. The *PCST1* widely expressed in Arabidopsis, including roots, axils of stem leaves, flowers (sepal, conductive tissue of the petal, thrum, anther and stigmas), and the top and basal parts of the siliquas. It mainly localized in cell membrane. The overexpression of *PCST1* enhanced the sensitivity to osmotic stress in *Arabidopsis* based on the germination rate. While expression of *PCST1* decreased, and the sensitivity to osmotic stress had no obvious change in Arabidopsis. Its molecular mechanism study showed, that PCST1 response to osmotic stress resistance by regulating the proline, betaine synthesis, as well as the expression of key genes *SOS, NCED, CIPK*. PCST1 is composed of 403 amino acids. The START conservative domain, a transmembrane structure, the phosphatidyl choline binding sites are contained in PCST1. It is localized in cytoplasmic membrane. The *PCST1* widely expressed in the root, leaf, flower and siliquas. NaCl and mannitol suppressed the expression of *PCST1* and PCST1 can negatively control action of *Arabidopsis* in the osmotic stress. PCST1 regulates the synthetic pathway of proline, betaine and the expression of *SOS, NCED* and *CIPK* in response to the osmotic stress resistance.

## Background

In the process of agricultural production, all kinds of drastic/harsh environment are important reasons directly affecting crop yield. Mining the critical stress-related genes is the key to improve crop yields^1^. High salt, drought and low temperature seriously affect crop growth, development, directly affecting crop production.^2^ Therefore, how to improve the tolerance of crops becomes a major problem that should be solved. In the long-term evolution of plants, a variety of mechanisms are formed to resist environmental stresses, including morphology, physiology and biochemistry adaptive mechanism. With the *Arabidopsis* genome sequencing completed, a large number of gene sequences accumulated in the database, while the functions of most of them are unknown. PCST1 (*At1g55960*) is one of possessing START domain protein family in *Arabidopsis*. The function of PCST1 is no clear yet.

The conserved START domain is 30 KDa and consists of approximately 200 amino acids. It belongs to a class of steroid regulatory protein and could combine and transport cholesterol to inner membrane of mitochondria.^3^ It is defined as a protein related to combine with lipids and sterols amino acid sequence.^4^ In animals, the ligand portion of START has been confirmed. START and metastatic lymph protein (MLN64) can combine with cholesterol,^5^ phosphatidylcholine can transport protein-binding phosphatidylcholine,^6^ carotenoid-binding protein can combine carotenoids in silkworm.^7^ In addition, a protein called CERT (nephritis syndrome antibody binding proteins) have been discovered recently, ceramide can be transported by the START domain of CERT.^8^

By using X-ray crystallography method, the structure of START domain has been determined, which belongs to three species of mammal proteins: PCTP, MLN64 and StarD4. On the basis of this structure, START domains are classified as super-helical fold proteins family and exist in everywhere of cell organism everywhere.^9^ Researchers speculated that the members of the START superfamily bind lipids and steroids, while other fold supercoiled families can interact with a wide range of metabolites and other molecules, such as antibiotics, RNA, and antigens.^10^ START domains may have conservative interactions with lipids and sterols in higher species such as animals and plants. The function of START domains in animals (e.g. StAR and PCTP) are sterols and phospholipids substances transportation and metabolism.

In *Arabidopsis*, there are 35 members in the START domain protein-family.^11^ The function of nine of them has been determined and they all contain HD ZLZ START domain, they participate in the process of cortex development regulation (ATML1),^12,13^ floral organ formation (PDF2),^13^ anthocyanin accumulation (ANL2, FWA),^10,14^ epidermal hair growth (GL2),^15^ adaxial and abaxial polarity (PHV, PHB, REV)^16–18^ and vascular bundle development (ATHB-8).^19^ Previous studies reported that SSMP (At3g13062),^11^ which contains START domain, related to salt resistance. The PCST1 which possessed the START domain in Arabidopsis had the highest homology with SSMP. It is consists of 403 amino acids and possessed phosphatidylcholine binding sites and transmembrane structure. The structural characteristic of PCST1 may affect the composition and permeability of cell membranes, and further involves in plant osmotic stress regulatory processes.

Osmotic stress is a condition that the plant cannot get enough water because of some adverse environmental factors. Common osmotic stress factors include salt damage, drought, etc. Osmotic adjustment substances, such as proline and betaine, can reduce plant damages caused by osmotic stress.

Salt stress is an important abiotic stress, which directly affects plant growth and development by osmotic stress, specific ion toxicity, ion imbalance, oxygen stress and interfering with absorption and transport of mineral nutrients.^20^ Salt stress results in changes in many biochemical processes in plants, including the accumulation of low molecular weight metabolite, such as amino acid and betaine. This mechanism is commonly referred as the regulatory mechanism of compatible solutes and ion transport.^21^ In addition, oxygen stress will occur simultaneously with salt stress. This phenomenon results from the production of reactive oxygen species (ROS),^22^ such as super oxide radical (O^2-^), hydrogen peroxide (H_2_O_2_), and hydroxyl radical (OH^−^). In plant cells, the produced ROS interacts with some of the important cellular molecules and metabolites, which result in a number of damaging processes that cause cell damage. Excessive accumulation of ROS may cause toxic reaction of plants, such as lipid peroxidation, protein degradation and DNA mutation. It is found that some plant species protect their cellular and subcellular systems by enhancing some enzyme activity to suppress the reactive oxygen species and free radicals. The enzymes contain superoxide dismutase, catalase, peroxidase, glutathione reductase, and polyphenol oxidase. Ascorbic acid and glutathione are also important in the process of scavenging reactive oxygen species and free radicals.^23–25^ SOS (Salt Overly Sensitive) pathway is a classical salt resistance signal pathway in previous studies.^26^ SOS showed a critical role in regulating the ion homeostasis and improving the sodium resistance of plants under salt stress.^27^

Under drought stress, plants need osmotic adjustment to prevent dehydration of plants to maintain photosynthesis and stomatal opening, enhance the growth of root and promote the absorption of water. Water channel protein is a membrane channel, which plays an important role in the control of water content in cells.^28–30^ Many endogenous hormones in plants involve in drought stress. They may lead to a large number of plant signal transduction.^28^ When plants are subjected to drought stress, stress signals are perceived firstly through the receptor system and then transmit stress stimulus signals to second messenger.^31^ As a stress signal, ABA plays a critical role in plant stress resistance, especially in drought resistance.^32^ ABA biosynthesis is the basis of ABA signal generation. ABA synthesis key genes of *NCED* (9-cis-epoxycarotenoid dioxygenase)^33^ and *CIPK* (CBL-interacting protein kinase)^34^ play an important roles in the regulation of drought stress.

Proline is one of the components of plant protein and widely existed in plants in free state. In drought, salinity and other stress conditions, a large number of proline accumulated in many plants.^35^ Proline is the cytoplasmic osmotic adjustment substance, in addition, the accumulation of proline plays an important role in stabilizing the structure of biological macromolecules, reducing the acidity of cells, releasing ammonia and regulating cell redox potential as an energy base. The content of proline in plants reflects the stress resistance of plants to a certain extent. Proline is a response signal on osmotic substances and salt stress.^36^ The Δ1- pyrroline-5-carboxylate synthetase (P5CS) is a key enzyme in proline biosynthesis,^37–39^ and proline dehydrogenase (PRODH) catalyze proline degradation. Stress induces proline accumulation in plants, depending on the increase of P5CS activity.^40^ Under drought, high salt and other stress conditions, betaine is a very large amount of accumulation of osmotic adjustment substances, which has a great effect on the resistance to adversity. Betaine is synthesized by the catalysis of choline (CMO) and betaine aldehyde dehydrogenase (BADH).^41^ The content of malondialdehyde (MDA) reflects the peroxidation degree of cell membrane lipid in plants.^42–44^ High content of MDA usually accompanied with high degree of cell membrane lipid peroxidation and serious cell membrane damage.^45,46^ In general, the stress conditions, such as high temperature, salinity, and strong light, tend to induce membrane lipid peroxidation.

Proteins that contain START conserved domain functioned on lipids transport and metabolism, signal transduction, transcription regulation or other processes. PCST1 has a distant homology relationship with the START gene family in *Arabidopsis*, while PCST1 showed a closer relationship with PCTP that belongs to START family gene in mammals. Reports about the function of protein only containing START domain are less. The studies on the function of this protein on plant growth regulation and development are significant on clarifying the position of START domain in evolution.

In this study, the method of biological information was used to analyze the structure characteristic of PCST1 in *Arabidopsis*. To explicit the function of PCST1 in *Arabidopsis* under osmotic stress, the overexpression of *PCST1* and deletion mutant were obtained and used for analysis of stress resistance, then the mechanism of PCST1 participating in abiotic stress regulation maybe further explicit in *Arabidopsis*.

## Materials and methods

### Plant materials and growth conditions

The *Arabidopsis thaliana* (L.) Heynh. ecotype Colombia (Col-0) was used as the WT and the genetic background for transgenic plants. The T-DNA insertion mutants of *PCST1* (SALK_053628, SALK_053637) were obtained from the Arabidopsis Biological Resource Center (ABRC), Columbus, OH, USA. Homozygous lines were generated from three generations for the analysis of segregation ratios. The deletion of gene in the mutant was determined by real-time reverse transcription-polymerase chain reaction (RT-PCR) using gene-specific primers and a left border T-DNA primer. The stable transformed plants were generated by floral dip method. Seeds were grown in Murashige and Skoog (MS) nutrient agar medium under continuous 70 μmol/(m^2^·s) light intensity at 22 ± 2°C. The basal MS medium was supplemented with 3% (w/v) sucrose, the mannitol or NaCl was added in treated groups. After transplanting *Arabidopsis* seedlings to soil (vermiculite: humus = 1:1), they were kept in a growth chamber with a photoperiod of 16 h/8 h (light/dark) at 22 ± 2°C. To analyze the expression of *PCST1* in response to osmotic stress, the 4-wk-old plants were treated with a solution of 150 mM NaCl or 300 mM mannitol. Plants treated with fresh water were used as controls. The tissues were collected at 1, 3, 6, 9, 12 and 24 h after treatment.

### Plasmid construction

Full-length cDNA of *PCST1* was amplified with the following primers containing restriction sites of *Bam*HI and *Sac*I: forward, 5′-GGATCCATGAAGAGCGTATCAACCTGGG-3′, reverse, 5′-GAGCTCTTAAATGGTGGTGGTCTGGGTG-5′. The amplified fragments were introduced into the T-vector PMD^TM^19 (TaKaRa Biomedicals, Dalian, China) and cloned into pSN1301. To create the *PCST1* pro::β-glucuronidase (GUS) construct, a 1.5-kb fragment upstream from the initiation codon was amplified using the forward primer with *Bam*HI and *Hin*dIII sites 5′-GGATCCTTGCTCTCGAGAATGAACCTTTTGA-3′ and reverse primer 5′-AAGCTTCGAATTCCAAAATCTCGACGATTTG-3′, and cloned into the pCAMBIA 1381z vector. The plasmid was electroporated into *Agrobacterium tumefaciens* GV3101 and transformed into Col-0 by the floral dip method. Transgenic plants were selected using 50 μg·ml^−1^ hygromycin. T3 or T4 homozygous lines were used for further experiments.

### Localization analysis

The full-length *PCST1* coding region was amplified with the primers containing *Sac*I and *Bam*HI sites (forward primer,5′-GAGCTCATGAAGAGCGTATCAACCTGGG −3′, reverse primer, 5′-GGATCCAATGGTGGTGGTCTGGGTG-3′). The PCR products were cleaved by *Sac*I/*Bam*HI and cloned into Cam35S-GFP (pCAMBIA2300-35S-GFP) to generate the *PCST1* expression plasmid. The plasmid (1.5 μg) was transformed into *Arabidopsis* and detected using a Laser scanning confocal microscope (FV1000IX81) with a wavelength of 488 nm.

### Stress tolerance assays

*Arabidopsis* seeds were sown on 1/2 MS solid medium with 200, 250, 300, 350 mM mannitol, or 100, 125, 150, 175 mM NaCl for 15 d. The germination rates, green cotyledon rates were counted, and the root length was measured.

### Expression analysis of genes related to proline and betaine biosynthesis

To analyze the expression of genes related to proline and betaine biosynthesis. *OE*, WT and *pcst1* plants were treated with 150 mM NaCl or 300 mM mannitol for 24 h. Seedlings grown under normal conditions were control. Realtime RT-PCR was performed to analyze gene expression levels (the gene names are listed in Table S1). The expression levels of genes in each type of plants (with or without stress treatment) were calculated.

### Real-time RT-PCR

Total RNA was isolated using the Trizol reagent and digested with DNaseI to remove DNA contamination. cDNA was synthesized using oligo dT as the primer. The genes of *18S rRNA and actin 3* were used as the internal controls. The primers used for RT-PCR are listed in Table S1. The reaction mixture (20 μl) contained 10 μl of SYBR Green Realtime PCR Master Mix (DBI®, Bioscience, Germany), 0.5 μM each of forward and reverse primers, and 1 μl of cDNA template (equivalent to 50 ng of total RNA). PCR was performed on an ABI 7500 system using the SYBR Premix Ex Taq^TM^(perfect real time) kit (TaKaRa Biomedicals, Dalian, China) under the following conditions: 94°C for 30s, 40 cycles of 94°C for 5s, 58°C for 15s, and 72°C for 10s, and 72°C for 7 min. Three biological replicates were performed. The data was calculated using the 2^−ΔΔCt^ method.^47^

### Water loss measurement

Eight leaves per individual mutant and WT plant growing in normal conditions for 4 week were excised. The leaves were kept on the laboratory bench at 22°C and weighed at the designated time intervals. Four replicates were performed for each line.

### Signaling pathway analysis

To analyze the role of *PCST1* in the stress resistance signaling pathway, WT, OE and *pcst1* mutant were treated with 150 mM NaCl and 300 mM mannitol and sampled at the designated time intervals. Total RNA of samples were extracted and reversed transcribed into cDNA. Real-time PCR was performed to analyze the expression of NaCl stress related gene *SOS1, SOS2, SOS3, HKT1* and mannitol stress-related genes *CIPK3, NCED3*. Primers are shown in Table S1.

### Statistical analysis

Data analysis was performed using SPSS 16.0 software (SPSS Inc, Chicago, IL, USA). ANOVA Tukey’s multiple comparison tests were used to exam significant differences (p < .05).

## Results

### Bioinformatic analysis of PCST1

**Figure 1.**
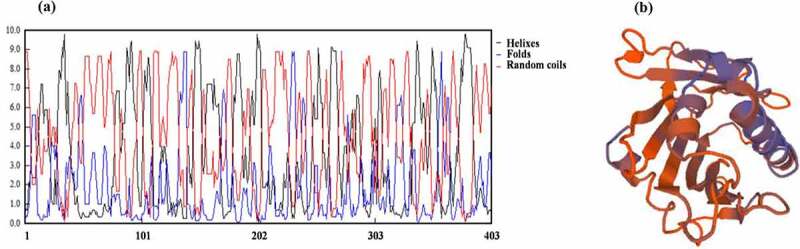
Bioinformatic analysis structures of PCST1. (a) Secondary structures of PCST1that consists of 148 helixes, 46 folds and 199 random coils. (b) Predicted tertiary structures of PCST1.

By consulting NCBI (National Center of Biotechnology Information) and TAIR (The Arabidopsis Information Resource) website, we know that PCST1 is a hydrophobin with 403 amino acids, the molecular weight is 45.3236 KDa, the isoelectric point is 9.75, only the START conservative domain (84–295) is contained. PCST1 possesses phosphatidylcholine binding sites. The function of PCST1 is unknown. Using TMHMM Server v. 2.0 to forecast the transmembrane structure of PCST1, it showed that the 21–43 amino acid position possesses the transmembrane region. Forecast PCST1 secondary structure, consists of 148 helixes, 46 folds and 199 random coils (Figure 1a). Tertiary structures of PCST1were predicted by SWISS – MODEL with homologous MODEL service software (Figure 1b). A 1.3-kb fragment received from TAIR upstream from the initiation codon was amplified, analysis PCST1 promoter contains components (Table 1), this study found 26 *cis*-element, participation in Arabidopsis response art defense, environmental stimuli, and hormone related components, including defense and stress response element (TC-rich repeats), drought induced MYB transcription factor binding sites (MBS); Gibberellin response element (P-box, GARE-motif) and auxin response elements (TGA-element); Anaerobic Response Element (Anaerobic Response Element, motorcycle), light Response Element (LAMP-Element, rbcS-CMA7a), etc. Which contains many relevant elements to the resistance to adversity, the response to the environmental stimulus and the hormone in the Arabidopsis. Show that *PCST1* may be involved in plant response to adversity defense.

**Table 1.** Promoter analysis of *PCST1.*

NAME	FUNCTION
AAGAA-motif	DNAse I footprinting
I-box	Part of a light responsive element
LAMP-element	Part of a light responsive element
AE-box	Part of a module for light response
ARE	Cis-acting regulatory element essential for the anaerobic induction
GCN4_motif	Cis-regulatory element involved in endosperm expression
Box 4	Part of a conserved DNA module involved in light responsiveness
CAAT-box	Common cis-acting element in promoter and enhancer regions
Sp1	Light responsive element
P-box	Gibberellin-responsive element
**MBS**	MYB binding site involved in drought-inducibility
Skn-1_motif	Cis-acting regulatory element required for endosperm expression
GTGGC-motif	Part of a light responsive element
TATA-box	Core promoter element around −30 of transcription start
**TC-rich repeats**	Cis-acting element involved in defense and stress responsiveness
circadian	Cis-acting regulatory element involved in circadian control
TGA-element	Auxin-responsive element
GARE-motif	Gibberellin-responsive element
GATA-motif	Part of a light responsive element
TCT-motif	Part of a light responsive element
Unnamed__1	Methylation protection
Unnamed__3	Methylation protection
Unnamed__4	Methylation protection
box – W1	Fungal elicitor responsive element
rbcS-CMA7a	Part of a light responsive element

### *Expression pattern of* PCST1 *in* Arabidopsis

**Figure 2.**
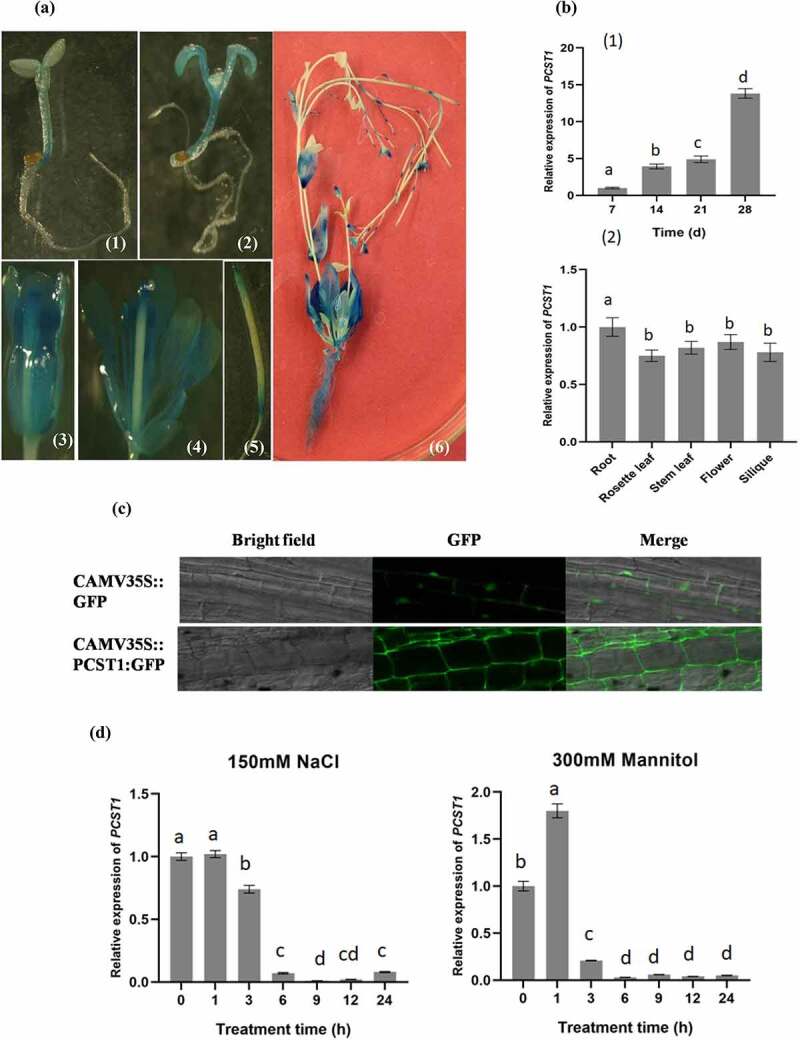
Expression pattern of *PCST1* in *Arabidopsis*. (a) Observation the GUS staining of *proPCST1::GUS* transgenic plants. (1) 5-d-old seeding; (2) 10-d-old seeding; (3) flower; (4) dissect of flower; (5) silique; (6) whole plant. (b) The expression of *PCST1* during different growth periods and different tissues. (1) Assay of the accumulation of *PCST1* transcript in different growth periods *Arabidopsis thaliana* plants. Total RNA was isolated from 7, 14, 21, 28-d-old wild-type (WT) plants; (2) Detection of *PCST1* transcript accumulation in different tissues of *Arabidopsis*. Total RNA was isolated from various tissues of 4-wk-old wild-type plants. Real-time reverse transcription-polymerase chain reaction (RT-PCR) quantifications were normalized to the expression of 18S rRNA. Values are means (±SE) of three replications. (c) Observation of location of PCST1-GFP under the laser confocal microscope. (d) Regulation of *PCST1* transcripts by NaCl and mannitol treatments. Assay of the accumulation of *PCST1* transcript in *Arabidopsis* in response to NaCl and mannitol treatments by real-time reverse transcription-polymerase chain reaction (RT-PCR). Wild-type were grown with sufficient water for 4 wk. The expression levels were normalized to that of *Actin 3*. Error bars represent standard errors (n = 3). Three independent replicates were performed. Statistical significance was determined by one-way ANOVA with Tukey’s post hoc test (p < .05).

To explore the expression pattern of *PCST1*, Transgenic plants *PCST1pro::GUS* were analyzed by using GUS staining (Figure 2a). *PCST1* was mainly expressed in hypocotyls, cotyledons and true leaves in seedlings, with more expression quantities in old parts than that in young parts. In mature strains, *PCST1* was widely expressed in roots, axils of stem leaf, the flowers (sepal, conducting tissue of the petal, thrum, anther and stigmas) and the top and basal parts of the siliquas. The temporal and spatial expression of *PCST1* in *Arabidopsis* was analyzed. The study demonstrated that expression of *PCST1* increased with time; *PCST1* expression was found in the root, rosette leaf, stem leaf, flower and siliqua of 6 weeks’ seedlings in *Arabidopsis* (Figure 2b). The subcellular localization of PCST1 was analyzed, showing that PCST1 was mainly localized in cell membrane (Figure 2c).

The 4 weeks’ old wild type was treated with 150 mM NaCl or 300 mM mannitol, leading to nearly 1/10 drop in expression of *PCST1* along the time to 6 h (Figure 2d). The result indicated that NaCl and mannitol suppressed the expression of *PCST1*.

### *Analysis of the expression of* PCST1 *in SALK and OE plants*

**Figure 3.**
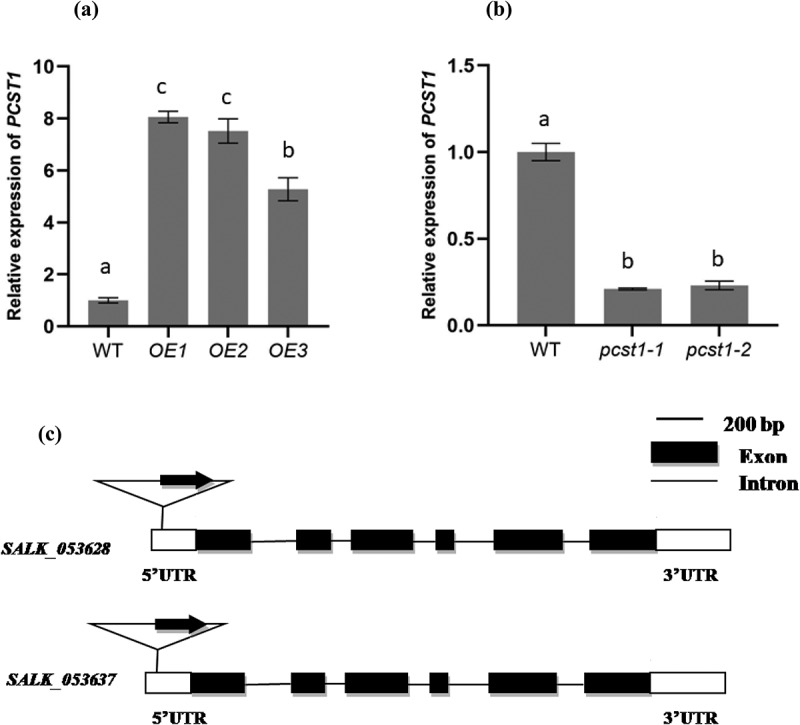
Analysis of the expression of *PCST1* in OE and SALK plants. The *Arabidopsis* plant with overexpression (a) and knockdown (b) of *PCST1* were analyzed using real-time PCR. The x-axis in each figure showed the line number, and 3 lines of overexpression (OE) and 2 lines of knockdown (pcst1) of *PCST1* were analyzed. The expression levels of *PCST1* were log2 transformed. Data are means ± SD from three independent experiments. (c) Schematic diagram of the T-DNA insertion site in the *PCST1* locus. Positions are shown for T-DNA insertions (triangle), exons (black rectangles), untranslated region (UTR) (white rectangles) and introns (lines). Data are means ± SD from three independent experiments. Statistical significance was determined by one-way ANOVA with Tukey’s post hoc test (p < .05).

We examined the expression of *PCST1* in mutant plants (SALK_053628, SALK_053637, *pcst1*) and overexpressing *PCST1* (OE) using real-time RT-PCR. The results showed that the expression of *PCST1* strongly increased in OE plants, and was downregulated more than 10-fold in SALK plants, compared with the control (Figure 3).

### *Overexpression of* PCST1 *enhanced NaCl and mannitol stress sensitivity in* Arabidopsis

**Figure 4.**
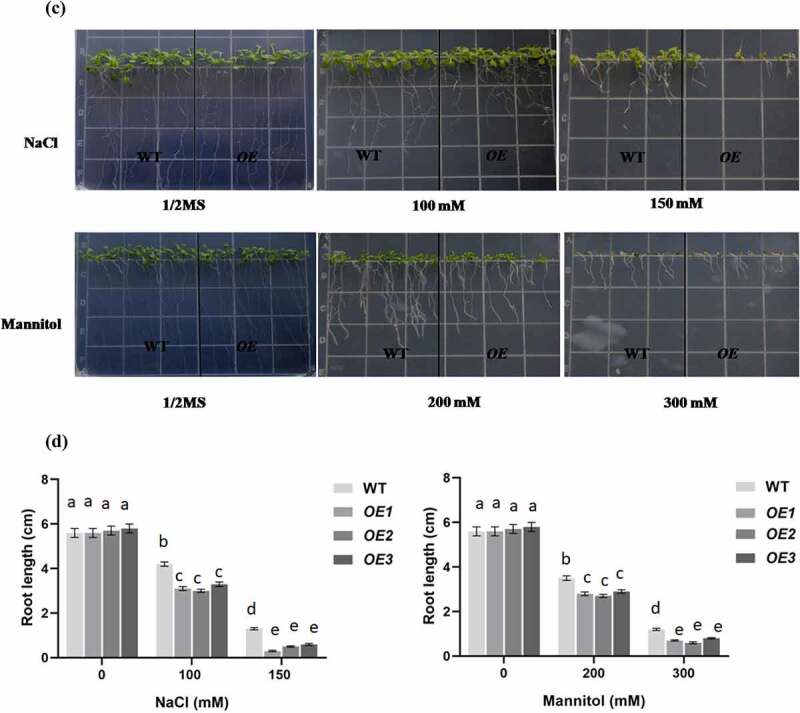
(continued)

To characterize the function of *PCST1* in stress tolerance, the types of plant with different *PCST1* expression levels were studied, including the following: WT plants; independent T3 homozygous transgenic plants which overexpressed *PCST1* (*OE*). After planting the wild type (Col-0) and *PCST1* transgenic seeds on the one-half-strength Murashige (MS) culture medium supplemented with/without 100, 150 mM NaCl or 200, 300 mM mannitol and culturing for 15 d. Under normal growth conditions, there were no differences in germination rate, green leaves rate, root length and growth phenotype among these plants (Figure 4). Under NaCl and mannitol treatment conditions, WT had significantly higher germination rate and green leaves rate than OE plants (Figure 4a,b). In addition, OE plants had significantly lower root growth rates than WT plants (Figure 4c). These results showed that overexpression of *PCST1* made an increased sensibility to osmotic stress.
**Figure 4.**
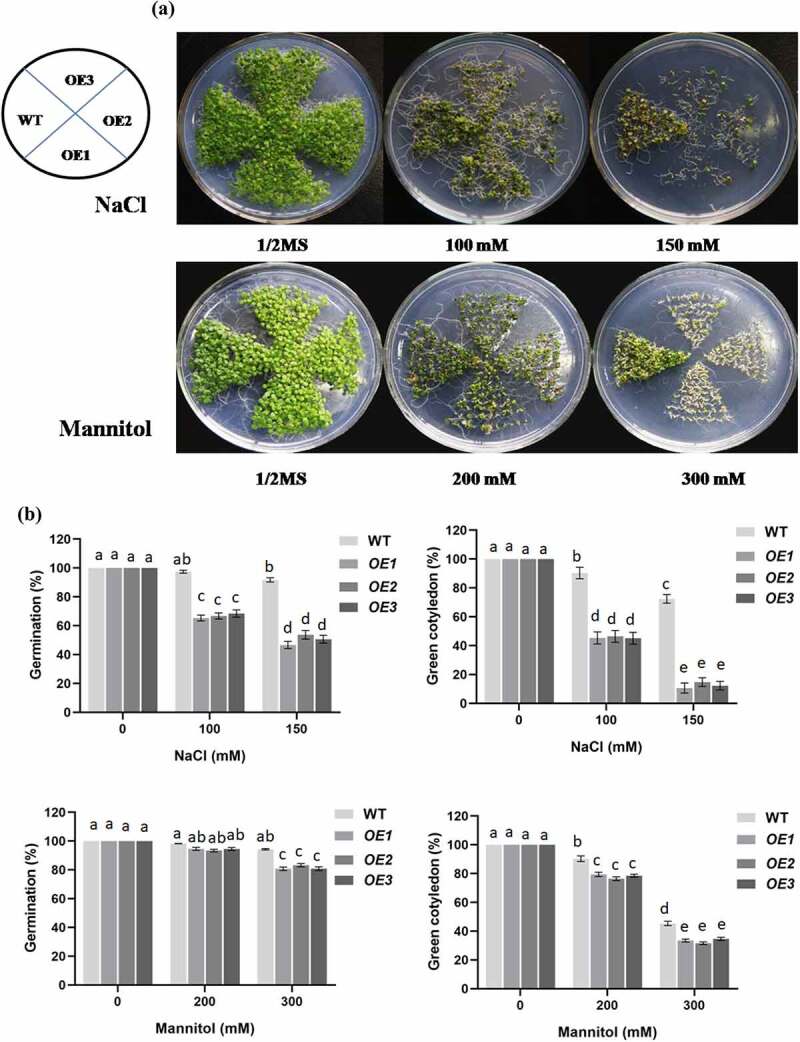
NaCl and mannitol responses of overexpression *PCST1* transgenic *Arabidopsis* during germination and post-germination growth. (a) Growth of WT and *OE* seedlings on Murashige and Skoog (MS) medium containing 100, 150 mM NaCl and 200, 300 mM Mannitol. Seeds were germinated and grown for 15 d. The photographs show representative seedlings. (b) Effects of NaCl and mannitol on the germination and green leave rates of WT and *OE* plants. Error bars represent the standard errors of *c*. 200 seeds from three independent experiments. (c) NaCl and mannitol effects on the growth of germinated WT and *OE* seedlings. Seeds of different genotypes were grown in a vertical position on MS medium containing 100, 150 mM NaCl and 200, 300 mM Mannitol. The photographs show representative seedlings. (d) Root length of WT and *OE* plants in response to NaCl and mannitol. Error bars represent the standard errors of *c*. 60 plants from three independent experiments. Statistical significance was determined by one-way ANOVA with Tukey’s post hoc test (p < .05).

### PCST1 *mutants showed no obvious response to osmotic stress of transgenic plants*

**Figure 5.**
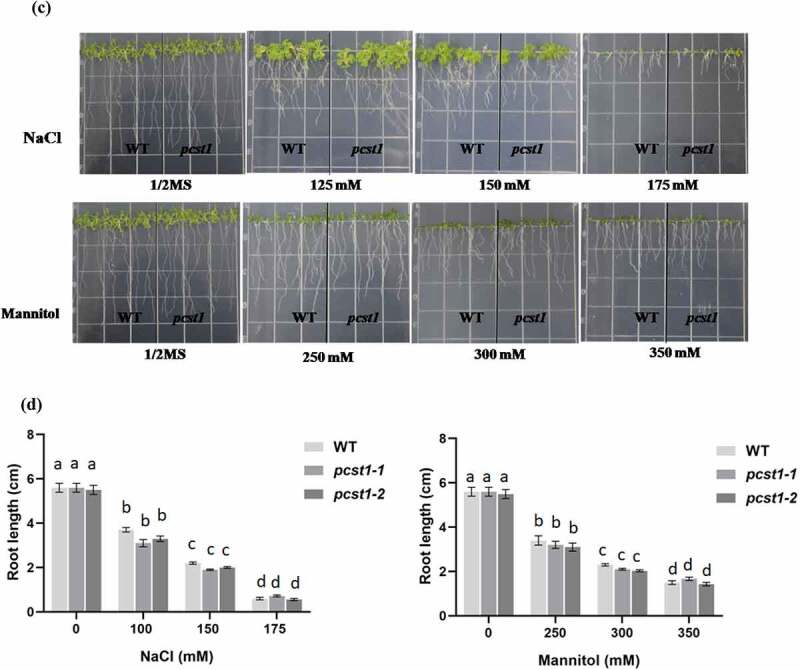
(continued)

Three types of plant with different *PCST1* expression levels were studied, including the following: WT plants, *PCST1* mutant plants (SALK_053628, *pcst1-1*; SALK_053637, *pcst1-2*). After planting the wild type and *PCST1* mutants seeds on the one-half-strength Murashige (MS) culture medium supplemented with 125, 150, 175 mM NaCl or 250, 300, 350 mM mannitol and culturing for 15 d. Under normal growth conditions, there were no differences in germination rate, green cotyledon rate, root length and growth phenotype among these plants (Figure 5). Under NaCl and mannitol treatment conditions, *pcst1-1* and *pcst1-2* plants showed no obvious response to them and had the same phenotype with the wild type (Figure 5a,b). In addition, WT, *pcst1-1* and *pcst1-2* plants had generally similar root growth rates (Figure 5c). The results showed that *PCST1* mutants had no obvious response to sensibility to osmotic stress.
**Figure 5.**
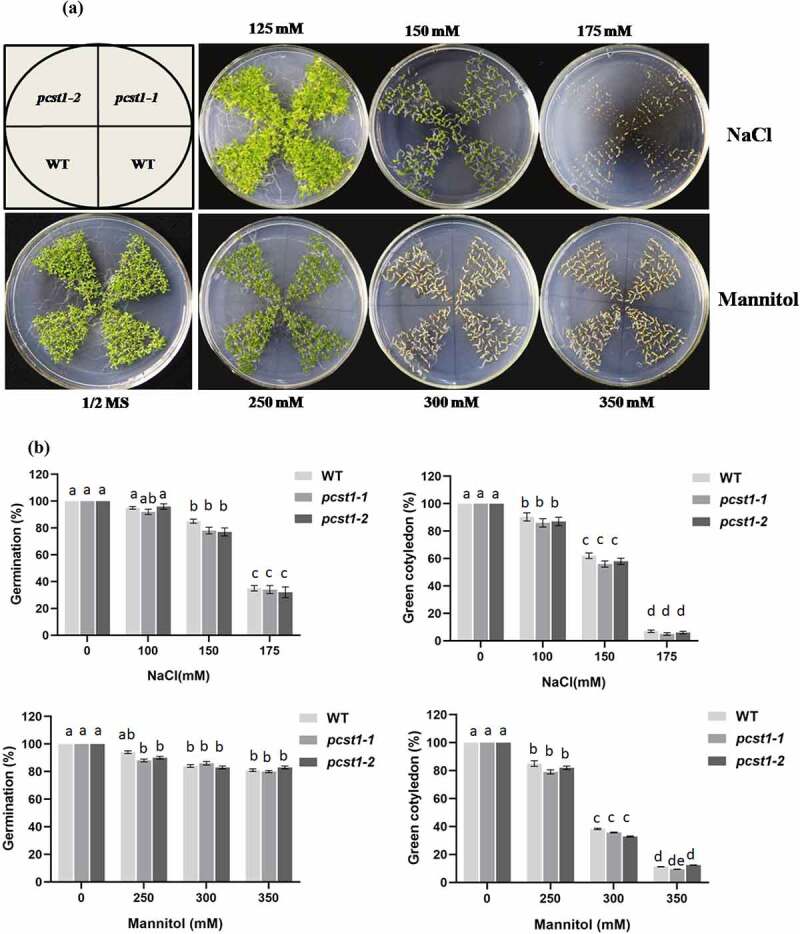
*PCST1* mutant plants are no obvious response to sensibility to salt and mannitol stresses. (a) Growth phenotype of WT and *pcst1* mutants in media containing 125, 150, 175 mM NaCl and 250, 300, 350 mM mannitol. Seeds were germinated and grown for 15 d. The photographs show representative seedlings. (b) Effects of NaCl and mannitol on the germination and greening leave rate of WT and *pcst1*. Error bars represent the standard errors of *c*. 200 seeds from three independent experiments. (c) Seedling root length of WT and *pcst1* mutants as affected by NaCl and mannitol. (d) The data analysis was the same as in **(b)**. Statistical significance was determined by one-way ANOVA with Tukey’s post hoc test (p < .05).

### PCST1 *confers stress tolerance through suppress of proline and betaine but improves water loss rate and Malondialdehyde accumulation*

**Figure 6.**
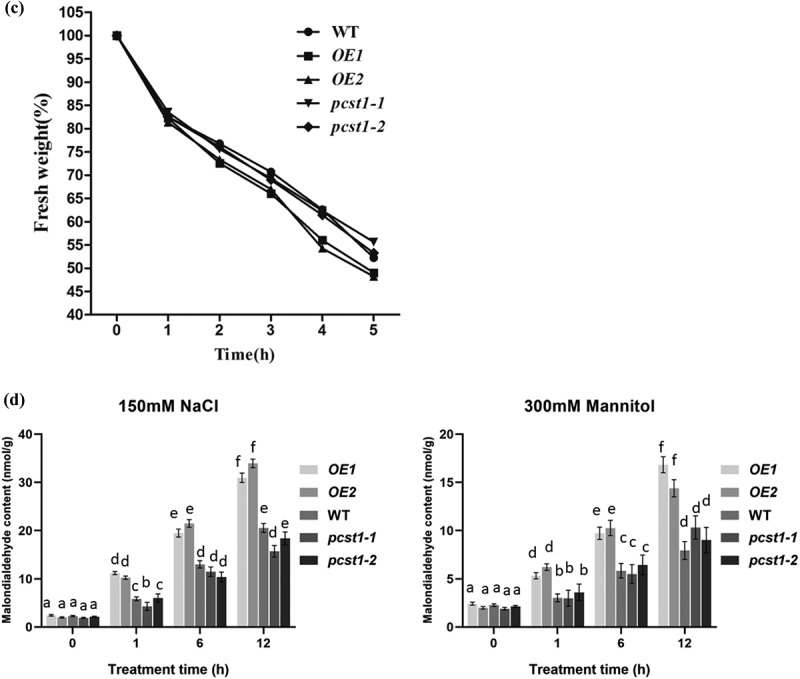
(continued)

Proline acts as an osmotic agent and radical scavenger to protect cells from damage caused by osmotic stress, and also helps plants to maintain sustainable growth under long-term stress conditions. As proline is quite important for osmotic stress tolerance, we examined whether *PCST1* is involved in proline accumulation under osmotic stress conditions. The proline contents among *OE*, WT and *pcst1* under salt and mannitol treatment conditions were significantly different, while there was no significant difference in proline content in these types of plants under normal growth conditions. The proline contents were lowest in *OE1* and *OE2*, whereas *pcst1-1* and *pcst1-2* had generally similar proline contents with WT (Figure 6a). We further examined the expression of proline biosynthesis- and degradation-related genes, including *P5CS* (At3g55610), *PRODH* (At4g34590). Under NaCl or mannitol treatment conditions, the expression of these genes was significantly different. The expression of *P5CS* was highest in WT plants and *pcst1-1, pcst1-2* plants, followed by *OE1* and *OE2*. In contrast, under NaCl and mannitol stress conditions, the expression of *PRODH* was highest in both *OE* lines, whereas it was lowest in WT and *pcst1*.
**Figure 6.**
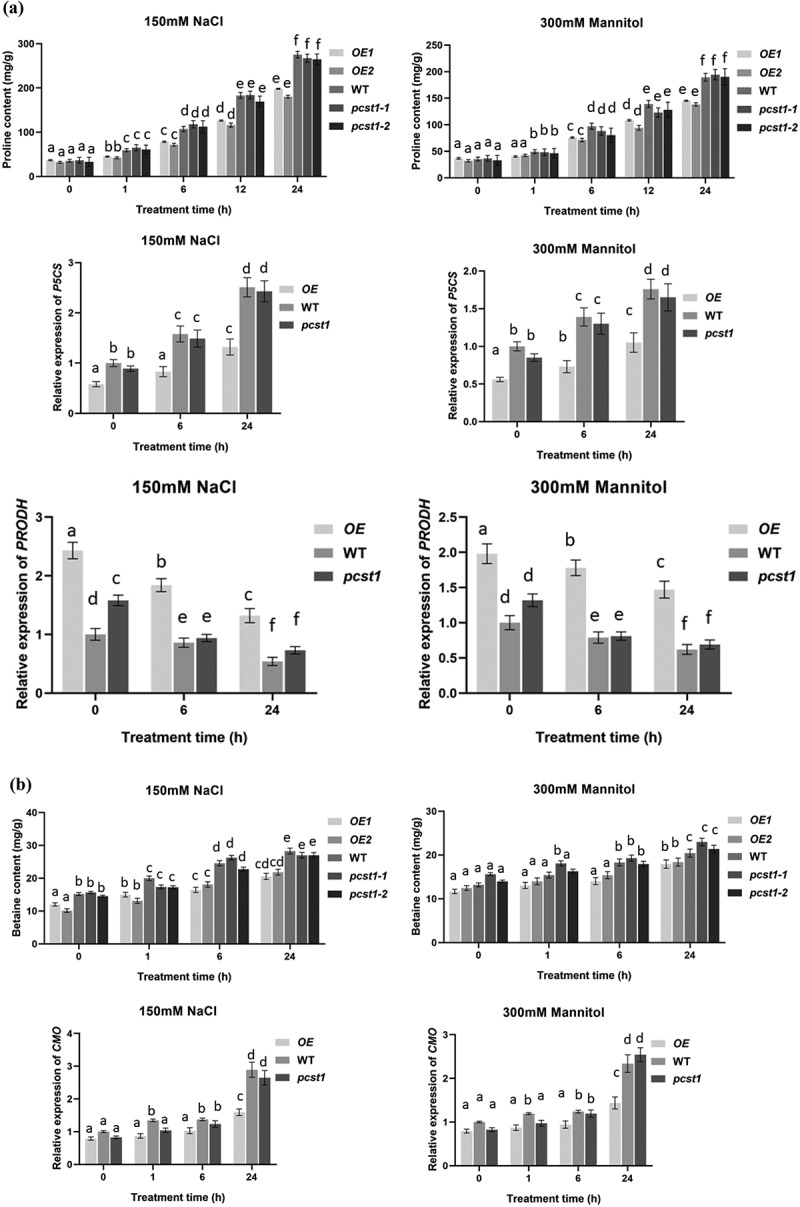
*PCST1* confers stress tolerance through suppress of proline and betaine but improves water loss rate and Malondialdehyde accumulation. (a) Proline biosynthesis regulated by *PCST1*. Analysis of proline level in *OE*, WT and *pcst1* and the expression of proline biosynthesis (*P5CS*) and degradation-related (*PRODH*) genes. The plants were treated with NaCl, mannitol for 1, 6, 12 and 24 h, and plants grown under normal conditions were harvested at the corresponding time points as controls. The relative expression level was calculated as the transcript level of a gene under NaCl or mannitol treatment for 6 or 24 h divided by the transcript level of that gene under normal conditions at the corresponding time point. Data are means ±SD from three independent experiments. (b) Analysis of betaine level in *OE*, WT and *pcst1* and the expression of betaine biosynthesis (*CMO*) genes. The plants were treated with NaCl, mannitol for 1, 6 and 24 h, and plants grown under normal conditions were harvested at the corresponding time points as controls. The relative expression level was calculated as the transcript level of a gene under NaCl or mannitol treatment for 6 or 24 h divided by the transcript level of that gene under normal conditions at the corresponding time point. Data are means ±SD from three independent experiments. (c) Water loss from detached leaves of WT, *OE, pcst1* transgenic plants. Water loss was expressed as the percentage of initial fresh weight. Values are means from eight leaves for each of five independent experiments. (d) Malondialdehyde content regulated by *PCST1*. Analysis of malondialdehyde level in *OE*, WT and *pcst1*. The plants were treated with NaCl, mannitol for 1, 6 and 12 h. Data are means ±SD from three independent experiments. Statistical significance was determined by one-way ANOVA with Tukey’s post hoc test (p < .05).

Betaine is a very large amount of accumulation of osmotic adjustment substances, which has a great effect on the resistance to adversity. There was no significant difference in betaine content among *OE*, WT and *pcst1* under normal growth conditions; on salt and mannitol treatment, the betaine contents in these types of plant were significantly different. The betaine contents were lowest in *OE1* and *OE2*, whereas *pcst1*and WT had generally similar betaine contents (Figure 6b). We further examined the expression of betaine that synthesized by *CMO*. Under salt or mannitol treatment, the expression of these genes was significantly different. The expression of *CMO* was highest in WT and *pcst1. CMO* was downregulated in *OE*, compared with the WT. Overexpression of *PCST1* improved water loss rate response to osmotic stress (Figure 6c). For water loss rate treatment, the leaves of 4-wk-old WT, *OE* and *pcst1* lines were weighed immediately (fresh weight, FW), kept on the laboratory beach at 25°C and weighed at the designated time intervals. Water loss was represented as the percentage of initial fresh weight at each time point. *OE1* and *OE2* had higher fresh weight decrease rate than WT and *pcst1*, whereas WT, *pcst1-1* and*pcst1-2* had generally similar fresh weight decrease rate. The results showed that the water holding capacity of *OE* strain was weakest, and the lowest stress tolerance. Overexpression of *PCST1* improved Malondialdehyde accumulation (Figure 6d). The content of MDA is the embodiment of the plant cell membrane lipid peroxidation degree of expression. There was no significant difference in MDA content among *OE*, WT and *pcst1* under normal growth conditions; on salt and mannitol treatment, the MDA contents in these types of plant were significantly different. The MDA contents were highest in *OE1* and *OE2*, whereas*pcst1*and WT had generally similar MDA contents.

### PCST1 *had an effect on genes of adversity resistance signal pathways*

Under the treatment of osmotic stress, the samples were taken at the designated time intervals. The qRT-PCR was carried out for detecting the expression of NaCl stress related gene *SOS1, SOS2, SOS3* and *HKT1*, and mannitol stress related gene *CIPK3, NCED3.*

**Figure 7.**
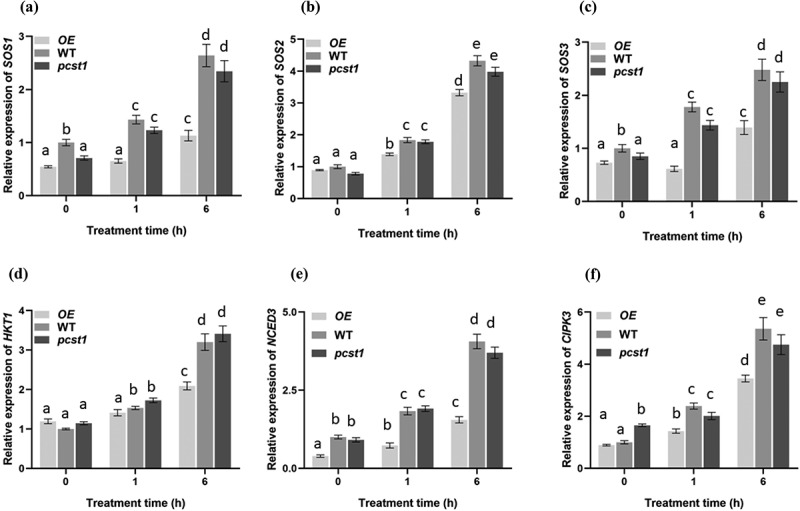
Analysis of the expression of NaCl stress related gene *SOS1, SOS2, SOS3* and *HKT1*, and mannitol stress related gene *CIPK3, NCED3* regulated by *PCST1*. Analysis of relative expression level in *OE*, WT and *pcst1*. The plants were treated with NaCl, mannitol for 1and 6 h. The expression of (a) *SOS1*, (b) *SOS2*, (c) *SOS3*, (d) *HKT1*, (e) *CIPK3*, (f) *NCED3*. Data are means ±SD from three independent experiments. Statistical significance was determined by one-way ANOVA with Tukey’s post hoc test (p < .05).

Under normal growth conditions, there were no differences in those genes among these plants (Figure 7). Under osmotic stress treatment conditions, those genes in WT had significantly higher expression than *OE* plants lines, whereas *pcst1* plants lines showed no obvious response to it and had the same expression with the wild type.

## Discussion

### *Expression patterns of* PCST1 *in* Arabidopsis

PCST1 have the homology to PCTP^11^ protein which contained mammalian START domain . Studies have shown that, PCTP can be combined with phosphatidylcholine,^48^ whose functions are transporting and metabolizing sterols and phospholipids substances. Due to PCST1 also has a phosphatidylcholine-binding site and phosphatidylcholine is a major component of phospholipids and phospholipid metabolism has a close relationship to cell membrane permeability and fluidity. At present, the protein only containing START domain in the process of plant stress resistance were rarely reported, SSMP only contains START domain and participates in the process of stress tolerance in Arabidopsis transmembrane protein, but PCST1 consisting transmembrene structure has nearly 70% of homology to SSMP. Bioinformatics analyzes defense-related stress tolerance response elements of *PCST1* upstream promoter sequence contained (TC-rich repeats) and MYB transcription factor binding sites (MBS), and the presence of these elements prompted that *PCST1* may be associated with defense response resilience, this is consistent results of this study, scilicet PCST1are involved in the regulation process under osmotic stress in *Arabidopsis*.

In this study, by analyzing the tissue localization of *PCST1* in Arabidopsis, *PCST1* in the whole growing cycle of growing from just two cotyledons to bear fruit in Arabidopsis were expressed. Seedling period is mainly composed of hypocotyl gradually to new site expression, from hypocotyl to cotyledon and true leaves, older parts more than young site expression; Adult seedling were expressed in the root, the Indus leaves, cauline leaf axil, stem, flowers (sepals, filaments, anther, stigma), pods. By analyzing the expression of partial distribution and growth period in PCST1 of Arabidopsis, we found that with the increase of plant growth days, *PCST1* expression quantity increased significantly, were expressed in various organizations, fully meet the GUS staining. Subcellular localization of PCST1 orientation on the cytoplasmic membrane, confirmed the bioinformatics analysis of PCST1 across a membrane structure.

### *PCST1 regulate the molecular mechanism of osmotic stress in* Arabidopsis

Studies have reported that the excessive Na^+^ ion in the soil caused imbalance in the body, moisture deficiency and ion toxicity,^49^ so some plants formed a Na^+^ efflux and Na^+^ segment processing, therefore they can maintain low intracellular Na^+^ concentration to adapt to the effects of salt stress on plant’s growth and development. SOS pathway in previous research is more classic salt signaling pathway.^26^ SOS3 and SOS2 located in the cytoplasm regulate SOS1 (plasma membrane Na^+^/H^+^ antiporter) on the cytoplasmic membrane, while the high-affinity potassium transporter HKT (high-affinity potassium transporters) and Na^+^/H^+^ antiporter NHX1 control the K^+^, Na^+^ regulation.^50^ So it will achieve intracellular balance of K^+^, Na^+^. Under drought stress, plants need osmotic adjustment to prevent plant dehydration phenomenon, maintain photosynthesis and stomatal opening, enhance root growth and promote the absorption of water. The water film is aquaporin channel. It plays an important role^28–30^ in terms of water content control cells.

Drought stress signaling pathways are divided into two kinds: dependent ABA and ABA not relying on two ways.^51^ ABA, as a stress signal in particular, plays an important role in drought resistance in plants.^33,52^ Since the ABA biosynthesis is the fundamental basis for ABA signal, ABA generates the biosynthetic NCED,^33^ and CIPK^34^ which plays an important role on the regulation of drought stress. Osmolytes proline and betaine play important roles in the regulation of plant salt and drought. Under drought, salinity and other stress conditions, many plants have accumulated much proline.^35^ Proline content in plants depends P5CS activity, and to some extent reflects the resistance of plants. P5CS is a key enzyme in the biosynthesis of proline, and PRODH catalyzed the proline degradation.^40^Betaine, as osmotic adjustment substance, which has much accumulation, has a very large role in resistance to stress . Betaine synthesis by CMO catalytic synthesis.^41^ MDA is a plant cell membrane lipid peroxidation degree of expression, and the high content of MDA, indicates higher plant plasma membrane lipid peroxidation, meanwhile the cell membrane was serious injury.^45,46^

**Figure 8.**
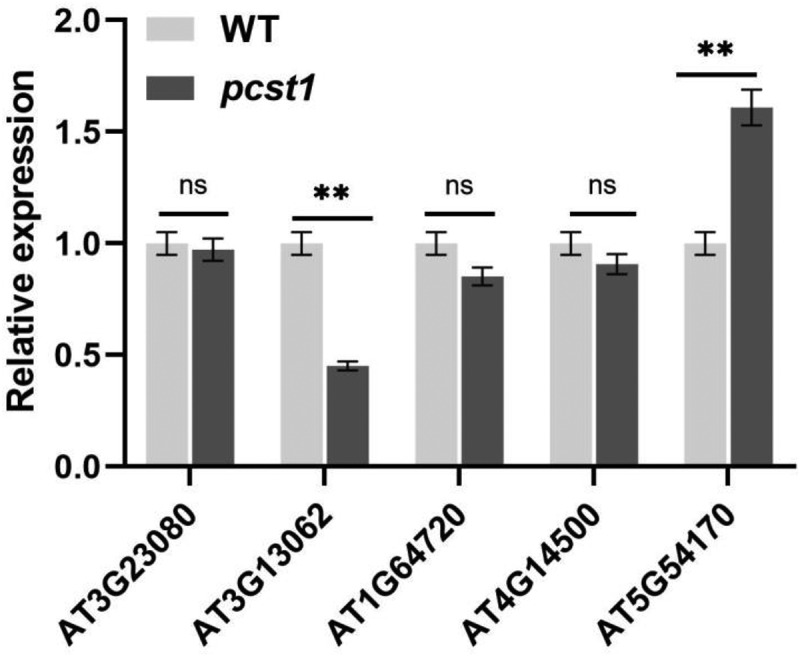
Analysis only contains the START structure genes expressed in *pcst1*. Statistical significance was determined by Student’s t-test (two-tailed) and are expressed as mean ± s.d. ns > 0.05, **p < .01.

This study through the *PCST1* function of gain and loss of transgenic Arabidopsis response to the stress of NaCl, mannitol, its various strains seedling germination rate, the rate of leaves and root length of phenotype observation and data statistics, and to analyze the phenotype of sprout, found expression strain sensitivity to osmotic stress increases, the T-DNA insertion mutants of osmotic stress sensitivity is not obvious; To express under osmotic stress, wild type and mutant Arabidopsis proline, betaine, malondialdehyde content determination, found expression strain proline, betaine content significantly lower than the wild type, MDA content is significantly higher than the wild type, while the mutant and wild type difference is not obvious. The expression analysis of osmotic stress gene *SOS1, SOS2, SOS3, HKT1, CIPK3, NCED3* showed overexpression of these genes expression in *PCST1* is significantly lower than the wild-type plant, and the difference between wild type and mutant plant is not obvious. We analyzed under osmotic stress, on the one hand, due to the excessive expression of *PCST1* through regulating effect on the key enzymes of osmotic regulation substances that affect the accumulation of osmotic regulation substances and the sensitivity to osmotic stress. On the other hand, because *PCST1* excess expression suppresses the osmotic stress signaling pathways of gene expression, the ionic imbalance inside and outside the cells causes more sensitivity to osmotic stress. *PCST1* T-DNA insertion mutants of Arabidopsis to osmotic stress sensitivity is not obvious due to redundancy of genetic function of family, we analyzed the expression of other gene that only contains the START structure domain in *PCST1* (Figure 8), found that in *pcst1, AT3G13062* and *AT5G54170* express obvious changes have taken place, the next step we will analyze whether *pcst1* due to genetic redundancy is not sensitive to osmotic stress, for each pair of interacting proteins is PCST1 screening at the same time, improve the *PCST1* regulatory mechanism of osmotic stress.

**Figure 9.**
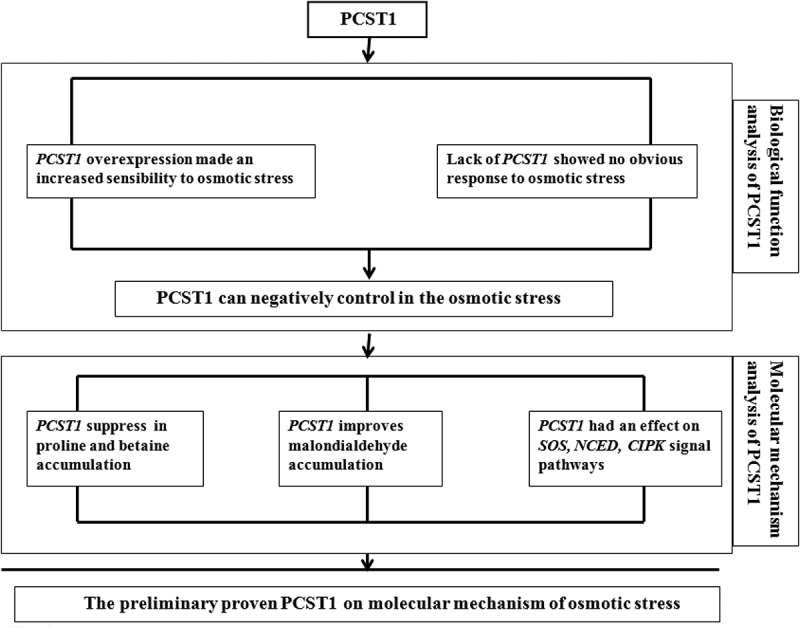
Analysis of the molecular mechanism of osmotic stress regulated by *PCST1.*

The results drawn from this study *PCST1* regulation sketch (Figure 9), preliminarily prove the molecular mechanism of osmotic stress regulation of PCST1 in Arabidopsis.

## Conclusion

PCST1 is composed of 403 amino acids, containing only START conservative domain, across a membrane structure, and the phosphatidyl choline binding sites, Positioning on the cytoplasmic membrane, the root, leaf, flower and fruit pods are expressed in the organization, in the expression is more mature organizations. NaCl and mannitol suppress the expression of *PCST1*. And PCST1 can negatively control in the osmotic stress action of Arabidopsis. PCST1 regulates the synthetic pathway of proline, betaine and the expression of *SOS, NCED* and *CIPK* for responding the osmotic stress resistance.

## Supplementary Material

Supplemental Material
